# Editorial: Unifying Insights into the Desiccation Tolerance Mechanisms of Resurrection Plants and Seeds

**DOI:** 10.3389/fpls.2020.01089

**Published:** 2020-07-21

**Authors:** Jill M. Farrant, John P. Moore, Henk W. M. Hilhorst

**Affiliations:** ^1^Department of Molecular and Cell Biology, University of Cape Town, Cape Town, South Africa; ^2^Department of Viticulture and Oenology, Faculty of AgriSciences, Institute for Wine Biotechnology, Stellenbosch University, Matieland, South Africa; ^3^Laboratory of Plant Physiology, Wageningen University and Research, Wageningen, Netherlands

**Keywords:** desiccation tolerance, homoiochlorophyllous, longevity, priming, senescence, subcellular glasses, sugars

It has been seven years since the Frontiers in Plant Science SI entitled “Current advances and challenges in understanding plant desiccation tolerance”. There we noted a growing theme pointing out the similarities of mechanisms of desiccation tolerance (DT) in seeds and vegetative tissues of Angiosperms (termed resurrection plants), invoking the theory that the latter acquired tolerance by re-activating their innate seed specific genetic elements in their vegetative. In the light of this, we entitled the call for this follow up SI “Unifying Insights into the Desiccation Tolerance Mechanisms of Resurrection Plants and Seeds.”

Contributions towards this SI have included valuable research into potential mechanisms of vegetative DT, not only in Angiosperms but also in Bryophytes and Pteridophytes, making the term “resurrection plants” in the title nominally incorrect. Nevertheless the question of whether there are unifying features of DT, or not, remains valid in our quest to understand the phenomenon, particularly for potential applied uses such as in seed conservation (as argued by Ballasteros and Walters) and ultimate production of extremely drought tolerant crops (e.g. Hilhorst and Farrant, 2018). The papers in this SI also reflect the use of several different disciplines, not only the more (currently) followed routes in use of omics, but also physiology, biochemistry and biophysics which aid to contextualize omic studies and facilitate understanding of the phenomenon DT.

Since 2013, genomes of seven species displaying vegetative DT have been sequenced and accompanying omics studies during de- and rehydration of these species published (summarized in Oliver et al., 2020). It remains to be shown that these genomes contain a “footprint” of vegetative DT in comparison with desiccation sensitive species, since most of the latter produce desiccation tolerant seeds or spores. Yet, various omics studies are advancing our insight into mechanisms and regulation of DT in both tracheophytes and non-tracheophytes, the former being exemplified in the review by Liu et al. where the authors compare transcriptomes and metabolomes of two Gesneriaceae resurrection species. Their contribution underscores the view that resurrection species share “core” mechanisms for withstanding extreme water loss, but also employ species-specific features, even among closely related species. In light of the universality of DT among plant taxa, and indeed variation among seeds in precise mechanisms of DT, the view that DT in angiosperms evolved through rewiring of their seed genes is being questioned (Lyall et al., 2019; Pardo et al., 2020). Rather, seeds should be considered as a subset of desiccation tolerant entities, each being equipped with similar core mechanisms but with their specific variation on the DT theme. An example in this respect are sugars, considered core metabolites contributing to DT in all plant tissues. In particular elevated levels of sucrose and raffinose family oligosaccharides (RFOs) are thought to form cytoplasmic glasses upon desiccation, these stabilizing the subcellular milieu (Hoekstra et al., 2001). Liu et al. showed that while both *Haberlea rhodopensis* and *Boea hygrometrica* accumulated sucrose during desiccation, they displayed different patterns in raffinose accumulation. In the former high raffinose levels were maintained at all times, whereas in the latter raffinose accumulated only during dehydration. The authors propose a priming role for raffinose in *H. rhodopensis*. In a metabolomic study on the acquisition of DT in developing seeds of *Erythrina speciose*, Hell et al. also observed an early increase in raffinose content and establishment of DT, well before the major drop in relative water content (RWC). This also suggests a priming effect at relatively high RWCs. Here an equivalent increase in sucrose by the end of maturation, again suggesting glass stabilization by combined raffinose and sucrose. Concomitantly, significant increase in arabinose-containing polysaccharides, known cell wall plasticizers (Moore et al., 2013), was observed, pointing at a role for cell wall flexibility adapting to dehydration. This notion is supported by Chen et al. in their review on the role of the cell walls in DT angiosperms, which show varying degrees of flexibility, depending on their overall strategy for mechanical stabilization of tissues in the desiccated state. The authors used transcriptome data, focusing on cell wall enzymes, to predict wall metabolism associated with desiccation. This approach revealed the common presence of transcripts encoding genes for xyloglucan endotransglycosylases, pectin methylesterases, mannanases, laccases, and cellulose synthases. Given the variation among species in extent of wall folding, the nature of involvement of these is likely to also vary.

Photosynthesis is a major generator of reactive oxygen species and desiccation tolerant organisms have various mechanisms to minimise production and counteract the damaging effects thereof (reviewed inter alia in Oliver et al., 2020). Retention of chlorophyll and chloroplast organisation, along with adequate protection of this apparatus during dehydration (termed homoiochlorophylly), is common to all non-tracheophytes and among tracheophytes in species where periods between desiccation events are relatively frequent. Here, the “risk” of chlorophyll retention is outweighed by the advantage of rapid resumption of photosynthesis upon rehydration. It is in part confirmed by López-Pozo et al. in their study on DT and longevity in chlorophyll retaining spores of three fern species from habitats with different water availabilities. The authors found that species occurring in environments where water availability in the sporulation season was less frequent produced more tolerant spores, whereas an intermediate degree of tolerance and longevity was displayed in species from mostly in wet environments. Tolerant spores were also shown to have more stable cytoplasmic glasses, which likely contributed to their more prolonged longevity in the dry state.

Further support for both the “priming hypothesis” and environment-induced desiccation response is given by Cruz de Carvalho et al. who evaluated the role of colony morphology in four species of moss, one aquatic, two from moist terrestrial environments and a fourth xeric in nature. This study highlighted two aspects of interest in mosses. Firstly, not only constitutive protective measures are at play and that slower inducible responses are also necessary. Here rate of dehydration is important with xeric species depending on their colony morphology to slow down rate of water loss. Secondly, that slow dehydration may be beneficial, and it can be asked whether priming takes place in mosses? Moss species are phenotypically plastic in the context of forming colonies, cushions and mats in response to environmental desiccation and these strategies are clearly linked to creating a microbiome resilient to desiccation stress.

An area of research first brought to attention by Griffiths et al. (2014) in our previous SI, is that of how drought induced senescence is suppressed in resurrection plants. Using existing omics data on tissues that survive desiccation, the authors proposed that signals promoting the onset of senescence as typified in drought sensitive species, are blocked. An often unreported phenomenon is that most resurrection plants experience some degree of desiccation induced senescence, usually of older tissues/leaves. Radermacher et al. utilise this phenomenon to compare physiological and metabolic changes in surviving, and thus non-senescent tissues with those destined to senescence during desiccation, in the resurrection monocot *Xerophyta schlechteri*. The authors found both tissue types initiated processes associated with DT in this species, but that upon dehydration below 35% RWC, hallmarks of age-related senescence, as typified in Arabidopsis were evident in older tissues. A marked increase in methyl-salicylic acid, shown to positively regulate senescence in desiccation sensitive species under water deficit stress, occurred at contents below this, suggesting active regulation of drought enhanced age related senescence.

Late embryogenesis abundant (LEA) proteins have long been associated with DT, yet their precise functions are largely unknown. Silva Artur et al. explored the structural plasticity of six of these intrinsically disordered proteins (IDPs) shown to be induced during desiccation in *X. schlechteri*. A multidisciplinary approach revealed that the *in silico* predicted structural dynamics of the proteins are reflected in the magnitude of their protective properties, both *in vitro* and *in vivo*, as overexpression of the encoding genes resulted in improved stress tolerance of E-coli and *Arabidopsis thaliana*.

Plant germplasm conservation relies predominantly on the ability to store seeds at sub-zero temperatures, this being enabled by their DT. Yet longevity of seeds under such storage conditions varies among species. Ballesteros and Walters investigated the properties of subcellular glasses in pea and soybean seeds, which respectively display extended and short longevity under conventional storage conditions. The authors conclude that it is the fragility in the nature of glasses formed in soybean that contribute to its short storage lifespan.

In summary, the papers in this SI confirm the concept of a common set of core elements which are differently utilized (and embroidered upon) in different DT organisms to achieve the common goal of survival of desiccation. This suggests that the DT trait has followed a path of divergent evolution with an ancient core mechanism as starting point. A relatively new concept coming out of this SI is the role of priming in DT. Current evidence epitomised in the review by Liu et al. suggests variation among species as to the requirement for this. While this could be related to frequency of desiccation events and rate of drying among species (primed species being more “prepared”), the question remains as to whether this might be more universally evident and perhaps necessary in seedlings to prepare them for impending desiccation events.

## Author Contributions

Each author contributed toward the review within their areas of expertise. JF compiled the review.

## Conflict of Interest

The authors declare that the research was conducted in the absence of any commercial or financial relationships that could be construed as a potential conflict of interest.
